# Mitochondrial Oxidative Stress and Cell Death in Podocytopathies

**DOI:** 10.3390/biom12030403

**Published:** 2022-03-04

**Authors:** Yu-Ting Zhu, Cheng Wan, Ji-Hong Lin, Hans-Peter Hammes, Chun Zhang

**Affiliations:** 1 Department of Nephrology, Union Hospital, Tongji Medical College, Huazhong University of Science and Technology, Wuhan 430022, China; drzhuyuting@hust.edu.cn (Y.-T.Z.); stellarwane@126.com (C.W.); 2 5th Medical Department, Medical Faculty Mannheim, University of Heidelberg, D-68167 Mannheim, Germany; jihong.lin@medma.uni-heidelberg.de (J.-H.L.); hans-peter.hammes@medma.uni-heidelberg.de (H.-P.H.)

**Keywords:** podocytopathies, mitochondrial oxidative stress, reactive oxygen species (ROS), antioxidant defense, cell death

## Abstract

Podocytopathies are kidney diseases that are driven by podocyte injury with proteinuria and proteinuria-related symptoms as the main clinical presentations. Albeit podocytopathies are the major contributors to end-stage kidney disease, the underlying molecular mechanisms of podocyte injury remain to be elucidated. Mitochondrial oxidative stress is associated with kidney diseases, and increasing evidence suggests that oxidative stress plays a vital role in the pathogenesis of podocytopathies. Accumulating evidence has placed mitochondrial oxidative stress in the focus of cell death research. Excessive generated reactive oxygen species over antioxidant defense under pathological conditions lead to oxidative damage to cellular components and regulate cell death in the podocyte. Conversely, exogenous antioxidants can protect podocyte from cell death. This review provides an overview of the role of mitochondrial oxidative stress in podocytopathies and discusses its role in the cell death of the podocyte, aiming to identify the novel targets to improve the treatment of patients with podocytopathies.

## 1. Introduction

Podocytopathies are defined as kidney diseases that are driven by podocyte injury with proteinuria and proteinuria-related symptoms as the main clinical features [1]. The incidence of podocytopathies seems to be gradually rising and they are the leading cause of end-stage kidney disease around the world [1,2]. However, efficient therapies for podocytopathies are lacking and current treatment can only retard the progression of diseases. Podocytes are highly specialized epithelial cells that are located in the glomerulus and constitute the filtration barrier with a glomerular basement membrane (GBM) and endothelial cells [2,3]. The interdigitated foot processes and slit diaphragm of podocytes are elementary structures for the selective filtration function of the glomerulus [1]. Excessive stress and harmful stimuli are likely to cause podocyte injury, possibly even death, which is clinically characterized by proteinuria and pathologically characterized by podocyte foot process effacement (FPE), detachment, and loss [1,2]. Considering the poor proliferation capacity of the podocyte, excessive podocyte loss progressively aggravates podocyte damage and eventually leads to global glomerulosclerosis [2]. Understanding how such detrimental stress and stimuli cause podocyte injury can help us to advance our acknowledgement of the mechanisms underlying the occurrence and progression of podocytopathies.

Mitochondrial oxidative stress refers to disrupted redox homeostasis by the elevated generation of reactive oxygen species (ROS) and (or) declined antioxidant defense capacity [4,5]. Increasing evidence suggests that oxidative stress plays a vital role in the pathogenesis of podocytopathies [6,7,8,9]. The excessive accumulation of ROS causes damage to intracellular components and impairs the normal structure and function of podocytes [7,8,10,11,12]. Accumulating evidence has placed mitochondrial oxidative stress in the focus of cell death research [4,13]. Classic cell death includes apoptosis, necrosis, necroptosis, pyroptosis, and ferroptosis [14,15]. Under pathological conditions, when redundant ROS-induced damage is beyond the compensatory capacity of podocytes, cell death occurs [8,16,17,18]. Conversely, the application of exogenous antioxidants can protect podocytes from cell death and improve kidney function [8,19]. Hence, this review provides an overview of the role of mitochondrial oxidative stress in podocytopathies and discusses its role in the cell death of the podocyte, aiming to identify novel targets to improve the treatment of patients with podocytopathies.

## 2. Spectrum of Podocytopathies

Genetic factors and non-genetic factors, such as immune, infectious, metabolic, and hemodynamic factors, can cause damage to the podocyte [2]. Therefore, podocytopathies can be further divided into genetic and non-genetic podocytopathies on the basis of the causes.

Numerous genetic researches identified many susceptibility genes relevant to podocytopathies, which can be divided into podocyte genes and syndromal non-specific genes in terms of the types of cells that experience genetic variation [1]. For example, genetic variants in *PLCE1* and *WT1*, two podocyte-expressed genes, result in the arrested development of glomeruli and the onset of diffuse mesangial sclerosis [20,21]. *APOL1* podocytopathy is the best studied podocytopathy that is associated with genetic variants of susceptibility genes [1]. In Africans carrying a high frequency of *APOL1* alleles, the prevalence of chronic kidney disease (CKD) is up to 16% [22].

Non-genetic podocytopathies consist of kidney diseases of many distinct causes. Immune injury to the podocyte can induce the development of podocytopathies, such as IgA nephropathy (IgAN), lupus nephritis (LN), and membranous nephropathy (MN) [2]. Metabolic and hemodynamic abnormalities damage the podocyte as well. Long-term poor glucose control and the hemodynamic changes in diabetics contribute to diabetic nephropathy (DN) [1]. Elevated blood pressure and the accompanying hyperfiltration can also induce podocyte injury, which plays an important role in the pathogenesis of hypertensive nephropathy [23]. Podocytopathies caused by infections and nephrotoxic substances are not negligible, for instance, HIV-associated nephropathy and collapsing glomerulopathy induced by pamidronate [24,25].

## 3. Oxidative Stress in Podocytopathies

**Figure 1:**
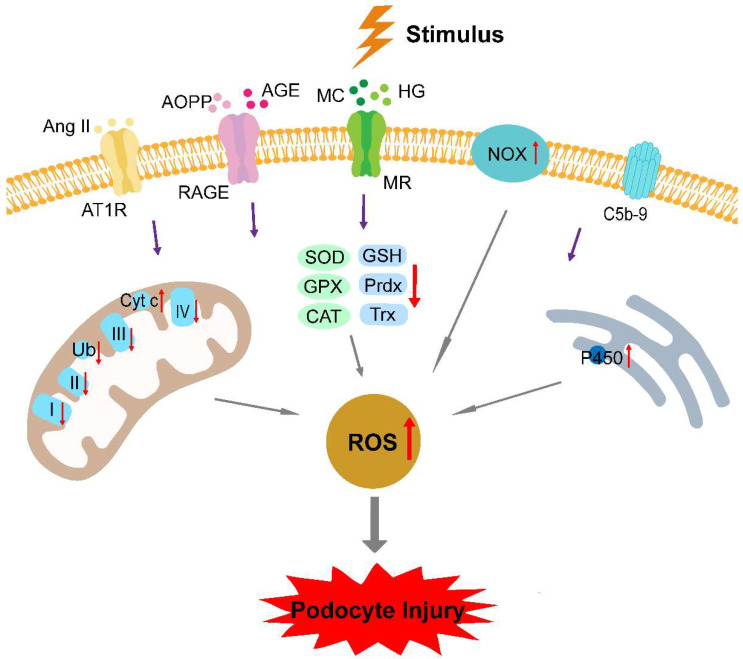
Oxidative stress plays a significant role in the pathogenesis of podocytopathies. Various harmful stimuli, such as puromycin aminonucleoside, immune complexes, HG, and Ang II, upregulate NOX, Cyt C, and P450, and downregulate mitochondrial respiration chain complexes (complex I, II, III, and IV), Ub, and antioxidant defense systems, including SOD, GPX, CAT, GSH, Prdx, and Trx. Excessive ROS accumulation in the podocyte causes damage to DNA, lipids, and proteins, and activates downstream signaling pathways, leading to podocyte foot process effacement, loss, and detachment, with clinical presentations of proteinuria and proteinuria-related symptoms. HG, high glucose; Ang II, angiotensin II; AT1R, angiotensin II type 1 receptor; AOPPs, advanced oxidation protein products; AGEs, advanced glycation end-products; RAGE, receptor of advanced glycation end-products; MC, mineralocorticoid; MR, mineralocorticoid receptor; C5b-9, C5b-9 membrane attack complex; NOX, NADPH oxidase; I, complex I; II, complex II; III, complex III; IV, complex IV; Ub, quinone; Cyt c, cytochrome c; SOD, superoxide dismutase; GPX, glutathione peroxidase; CAT, catalase; GSH, glutathione; Prdx, peroxiredoxin; Trx, thioredoxin; P450, cytochrome P450; and ROS, reactive oxygen species.

Under physiological conditions, a homeostasis between the production of ROS and the antioxidant defense system exists in the podocyte. ROS are a collection of chemical substances originated from incomplete reduced oxygen, which mainly consist of superoxide anion, hydrogen peroxide (H_2_O_2_), singlet oxygen, and hydroxyl radical [13,26]. In the podocyte, ROS mainly come from the mitochondrial respiration chain and NADPH oxidase (NOX) [27,28,29]. The mitochondrial respiration chain is mainly composed of NADH dehydrogenase (complex I), succinate dehydrogenase (complex II), ubiquinol-cytochrome c reductase (complex III), cytochrome c oxidase (complex IV), cytochrome c (Cyt C), and quinone [30,31]. Mitochondrial respiration chain dysfunction contributes to excessive ROS generation. Antioxidant defense systems are developed in the organism to eliminate ROS generated from various sources. Enzymatic defense systems include superoxide dismutase (SOD), glutathione peroxidase (GPX), catalase (CAT), and thioredoxin reductase (TrxR) [4,32]. Additionally, antioxidants are comprised of ascorbic acid (Vitamin C), α-tocopherol (Vitamin E), glutathione (GSH), thioredoxin, peroxiredoxin (Prdx), and carotenoids [4,32]. When the redox homeostasis is disrupted due to external stimuli under pathological conditions or inherent defects of podocyte, oxidative stress occurs and leads to podocyte injury and renal damage (Figure 1). Stimuli that are harmful to the podocyte, such as puromycin aminonucleoside (PA), high glucose (HG), and angiotensin II (Ang II), can all contribute to the intracellular accumulation of ROS [28,33,34].

In this section, we discuss and summarize the mechanisms by which ROS production is elevated in podocytopathies.

### 3.1. Elevated ROS Production

In focal segmental glomerulosclerosis (FSGS), the level of NOX4, a subunit of NOX complex, is higher in patients with steroid-resistant nephrotic syndrome (SRNS) than those with steroid-sensitive nephrotic syndrome, and the level of ROS is also higher in the glomeruli isolated from patients with SRNS [35]. The induction of NOX4 is observed in the PA-treated podocyte as well [27]. In the lmai rats, a spontaneous FSGS model, the activation of the angiotensin II type 1 receptor (AT1R) by Ang II leads to oxidative stress via upregulating the expression of NOX and downregulating the expression of Nrf2 [36]. In the FSGS phase of puromycin aminonucleoside nephrosis, cytochrome c oxidase subunit 1 (COX1) in the glomerulus is decreased [6]. Mice that lack the cytochrome c oxidase assembly cofactor heme A, farnesyltransferase (COX10), develop severe FSGS at an early age [37]. In the PA-treated podocyte, a decrease in COX1, 2, and 4, and a reduction in complexes I and IV are observed [27]. A mutation in *ADCK4*, which participates in the biosynthesis of coenzyme Q10 (CoQ10), causes the development of FSGS [38]. Whole-exome sequencing and Sanger sequencing suggests that a coenzyme Q10 mono-oxygenase 6 (COQ6) mutation might increase the production of ROS and associate with the occurrence of FSGS [39]. In PA-induced minimal change disease (MCD), CYP2B1, a cytochrome P450 isozyme, is a source of ROS and catalytic iron formation in the podocyte, with CYP2B1 inhibitors cimetidine and piperine alleviating PA-induced proteinuria and attenuating the increase in H_2_O_2_ and catalytic iron [33,40]. Adriamycin can induce the downregulation of complex I subunits both in vitro and in vivo [41]. In patients with a congenital nephrotic syndrome of the Finnish type, the kidney cortex shows the downregulation of complexes II and IV, and the mRNA levels of COX1 and COX2 are reduced [9,42,43].

For patients with IgAN, the copy numbers of COX3 and nicotinamide adenine dinucleotide dehydrogenase subunit 1 (ND1) in the urine are higher than the healthy controls [44]. After treatment, the changes in urinary COX3 and ND1 levels are positively associated with the changes in proteinuria and negatively associated with the changes in eGFR [44]. In the plasma of IgAN patients, the levels of advanced oxidation protein products (AOPPs) and malonaldehyde (MDA) are elevated, which can be attenuated by angiotensin-converting enzyme inhibitors (ACEIs) [7]. In the model of MN, complement activation in the podocyte reinforces the assembly of the C5b-9 membrane attack complex to induce the generation of ROS, which is mainly mediated via the upregulation of NOX [45,46,47]. An overexpression of CYP2B1 markedly elevates the generation of ROS and damages the cytoskeleton of the podocyte in MN, while silencing CYP2B1 ameliorates podocyte injury [48]. In podocytes treated with LN plasma, the production of ROS by mitochondria increases and the level of mitophagy decreases [49]. IgG from LN patients can elicit the generation of ROS as well [50].

HG-induced mitochondrial ROS generation is regarded as the main mechanism of DN [28,51]. Extracellular HG induces the formation of intracellular ROS in the podocyte via NOX and mitochondrial respiration [8,52,53]. In the kidney of Zucker obese rats, the expressions of NOX2, NOX4, and AT1R are upregulated [54]. Silencing NOX4 or pretreatment with NOX inhibitor GKT137831, diminishes high glucose-elicited ROS generation and the upregulation of profibrotic markers of the podocyte [55]. NOX5 is also upregulated in human diabetic kidney biopsies and type I diabetic mice [56,57]. Transgenic mice expressing podocyte-specific NOX5 develop albuminuria, podocyte FPE, and the elevation of systolic BP through the generation of ROS, which are further aggravated under streptozotocin (STZ)-induced diabetes [56]. Hyperglycemia can also activate the mineralocorticoid receptor (MR) to induce podocyte injury and proteinuria via the generation of ROS from NOX [58]. CYP4A, a member of the cytochrome P450 family, is upregulated and 20-hydroxyeicosatetraenoic acid is increased, followed by NOX activation [53]. In db/db mice and the HG-cultured podocyte, the activity of complexes I and III is reduced, while the functional expression of complex I in the cultured podocyte prevents the increase in mitochondrial ROS caused by HG [28,59]. In diabetic mice, the enhanced expression of p66Shc, a redox-regulating protein associated with Cyt C, promotes the generation of ROS from mitochondria [60,61].

A high-salt diet and Ang II both increase NADPH-dependent ROS formation in the kidney cortex [34,62]. Ang II induces an ROS-dependent rearrangement of the cytoskeleton to facilitate podocyte migration and eventual podocyte depletion, which can be inhibited by an NOX4 knockdown [12,63]. MR activation is involved in the podocyte damage in hypertensive kidney damage and metabolic syndrome as well [64,65]. Sprague–Dawley rats administrated with aldosterone and high salt display podocyte injury and proteinuria, resulting from elevated NOX activity and ROS production in the podocyte, while selective aldosterone blocker eplerenone and antioxidant tempol can protect against aldosterone-induced injury and proteinuria [64,65].

Homocysteine-induced podocyte injury and glomerulosclerosis involve NOX activation and endogenously generated superoxide anion and H_2_O_2_ that mediate the NLR family pyrin domain containing 3 (NLRP3) inflammasome formation [66]. In spontaneously hypertensive rats, a high level of homocysteine (Hcy) upregulates the mRNA levels of NOX2 and NOX4 [29]. AOPP has been shown to be closely associated with the severity of CKD and proteinuria [67]. It can bind to the receptor of advanced glycation end-products (RAGE) to induce NOX-mediated ROS generation, which further activates the Wnt/β-catenin pathway, resulting in FPE, matrix accumulation, and proteinuria [67,68,69]. Transforming growth factor β (TGFβ) has been shown to accumulate in renal diseases, and it can induce the upregulation of NOX4 via the Smad-dependent pathway and following ERK1/2-mTOR activation [70,71].

### 3.2. Deficient Antioxidant Defense Systems

Patients diagnosed as FSGS show markedly lower plasma and urinary GPX levels compared with MCD and healthy controls [72]. The glomerular GPX immunostaining score in FSGS is also lower than MCD and the normal controls both in patients and rats [72]. CoQ10 acts not only as an electron carrier, but also as an important antioxidant. In patients with idiopathic FSGS, CoQ10 partial deficiency might disturb podocyte biological function and induce renal lesions [73]. Proteinuria and podocyte FPE, resulting from podocyte lesions caused by a superoxide anion formed upon PA via xanthine oxidase, can be attenuated by intravenous SOD treatment [74]. Mitotempo, a SOD mimetic, reduces urinary protein excretion and lipid peroxidation via inhibiting oxidative stress and tissue damage in the MCD model [75]. In adriamycin-induced nephropathy, a model of FSGS, decreased activities of CAT, GPX, and SOD have a vital role in the occurrence of tubulointerstitial injury and glomerulosclerosis [27,76]. Mice lacking CAT are more vulnerable to the renal toxicity of adriamycin than wild mice, and manifest severe proteinuria and pathological lesions [77]. The supplementation of vitamin E in the diet of rats ameliorates adriamycin-induced renal injury via decreasing the activity of SOD1, SOD2, CAT, and GPX, both in the cortex and glomeruli [78].

In MN, anti-SOD2 IgG4 is detected in the plasma of patients, and it is co-localized with C5b-9 in the immune deposits, suggesting that SOD2 has an important role in the pathogenesis of MN [79]. A lower level of SOD in the plasma of IgAN patients is observed as well, which can be significantly reversed by treatment with ACEI [7]. IgAN patients treated with vitamin E show significantly lower proteinuria [80]. In the kidney of pristane-induced LN, the expressions of SOD1 and CAT are significantly reduced [81].

In the DN murine model and HG-treated podocyte, the expression levels of SOD2 and total SOD are significantly lower than controls [59,82,83]. Additionally, HG-induced oxidative stress-dependent podocyte loss and proteinuria can be attenuated by mitotempol [84]. The activities of CAT and GPX are lower at an early age of Zucker obese rats compared with controls, resulting in the accumulation of H_2_O_2_ [85]. HG treatment decreases the expression of Prdx6, an isoform of the Prdx family of antioxidant enzymes, which catalyze the reduction in H_2_O_2_ and ROS, but increases the expression of the thioredoxin-interacting protein (TxNIP) to inhibit the antioxidative function of Prdx and thioredoxin, leading to the accumulation of ROS and oxidative stress [17,86,87]. However, the overexpression of Prdx6 in the mouse podocyte restrains HG-induced ROS and MDA generation and recovers the activities of SOD and GSH [17]. In streptozotocin-induced TxNIP KO mice, albuminuria, serum creatinine, podocyte FPE and loss, GBM thickening, mesangial matrix expansion, and glomerulosclerosis are effectively attenuated, as well as oxidative stress and inflammation [86].

A high-salt diet and Ang II both downregulate the expression level of SOD in the kidney cortex [34]. Ang II stimulates the downregulation of Prdx2 in the podocyte, which results in elevated ROS release and protein peroxidation [88]. In spontaneously hypertensive rats administrated with high Hcy, a significant decrease in SOD and increase in MDA are observed [29].

Intracellular ROS excessive accumulation can induce damage to cellular macromolecules, such as DNA, proteins, and lipids, ultimately leading to podocyte injury, renal lesions, and the occurrence and progression of podocytopathies [7,10,11,32]. ROS-mediated nuclear and mitochondrial DNA damage and the upregulation of cell-cycle checkpoint proteins result in a poor proliferation of podocytes, which can be mitigated by radical scavenger 1,3-dimethyl-2-thiourea (DMTU) [10,89]. The generation and deposition of lipoperoxides, such as MDA and 4-hydroxynonenal (4-HNE) caused by ROS from damaged mitochondrion, are observed [7,43]. Additionally, elevated ROS also disturb the balance among key regulators of actin cytoskeletons, including RhoA and Rac1, resulting in actin cytoskeleton reorganization and subsequent podocyte FPE, depletion, and glomerulosclerosis [12,47,48]. Deglycosilation of alpha-dystroglycan in the glomeruli mediated by ROS leads to podocyte detachment from the GBM [11]. Moreover, ROS mediate the upregulation of profibrotic markers in the podocyte and promotes the inflammatory responses resulting in podocyte injury [55]. Pathological and ultrastructural features of podocytopathies, such as podocyte FPE, detachment, and loss, GBM thickening, mesangial matrix expansion, tubulointerstitial fibrosis, and glomerulosclerosis, as well as renal dysfunction indicators, such as urinary protein excretion and eGFR, are closely related to oxidative stress in the podocyte [8,44,56,67,68,69,90].

## 4. Oxidative Stress and Cell Death in Podocytopathies

Cell death is a highly conserved process that not only participates in the morphogenesis and development of organisms, but also the pathophysiological process. Different patterns of cell death manifest with discriminating morphological alterations and mediate the pathogenesis of various diseases [14]. Apoptosis is perhaps the most widely recognized pattern of cell death, which involves the activation of caspases (CASPs), especially CASP3, with morphological manifestations of cytoplasmic shrinkage, chromatin condensation, nuclear fragmentation, and ultimate apoptotic body formations [4,14,91]. Necrosis is another form of cell death characterized by the swelling of organelles and the whole cell coupling with negative morphological features of apoptosis and autophagy, and triggered by cyclophilin D-dependent permeability transition pore complex formation [14,92]. Necroptosis is generally manifested as a necrotic morphology and depends on the sequential activation of receptor interacting with serine/threonine kinase 3 (RIPK3) and a mixed lineage kinase domain, such as pseudokinase (MLKL) [14]. Pyroptosis is an inflammatory response depending on the activation of CASP1 and the formation of the plasma membrane pore by gasdermin-D (GSDMD), and manifests chromatin condensation that is different from apoptosis, cellular swelling, and plasma membrane permeabilization [14,91]. Ferroptosis is a newly discovered iron-dependent cell death that has gained significant attention, and is initiated by lipid peroxidation and manifests as necrotic morphology [14,93].

The death of the podocyte contributes to the progressive loss of the podocyte and subsequent glomerular filtration barrier destruction, and podocyte loss is the primary feature for the progression of podocytopathies [18,90,94,95]. In patients with DN, the number of podocytes in the kidney is decreased, and this is the strongest predictor of the progression of DN [96]. A large amount of research has confirmed that ROS play a significant role in regulating cell death [4,13,14]. Thus, in this section, we summarize the mechanisms by which the accumulation of intracellular ROS can regulate podocyte death.

Apoptosis has been the focus of extensive researches and is widely confirmed to be mediated mainly by two primary signaling pathways: the intrinsic apoptosis pathway and the extrinsic apoptosis pathway [14,97]. The intrinsic apoptosis pathway, or mitochondria-mediated pathway, can be activated by DNA damage, endoplasmic reticulum (ER) stress, oxidative stress, and other stimuli [14,97]. Proapoptotic members of the B-cell lymphoma-2 (BCL2) protein family, such as the BCL2-interacting mediator of cell death (BIM), BH3 interacting domain death agonist (BID), and BCL2 binding component 3 (BBC3), are activated transcriptionally or post-transcriptionally to induce the oligomerization of BCL2 associated X (BAX) and BCL2 antagonist/killer 1 (BAK) and the formation of mitochondrial outer-membrane permeabilization (MOMP) [14,97]. However, pro-survival members of the BCL2 protein family, such as BCL2 and BCL2-like 1 (BCL2L1), can antagonize MOMP to inhibit the occurrence of apoptosis [14,98]. MOMP facilitates the release of Cyt C, which can bind apoptotic peptidase activating factor 1 (APAF1) to mediate the activation of pro-CASP9 [14,97]. The extrinsic apoptosis pathway, or death receptor-mediated pathway, can be triggered by the Fas ligand (FasL), TNF-α, and the TNF-related apoptosis-inducing ligand (TRAIL) [97]. The death receptor executes the assembly of the death-inducing signaling complex (DISC), which regulates the activation of pro-CASP8 [97,99]. CASP9 from the intrinsic apoptosis pathway and CASP8 from the extrinsic apoptosis pathway can both cleave pro-CASP3 to activate CASP3, which cleaves cellular proteins leading to morphological damage and cell death [14,97].

**Figure 2:**
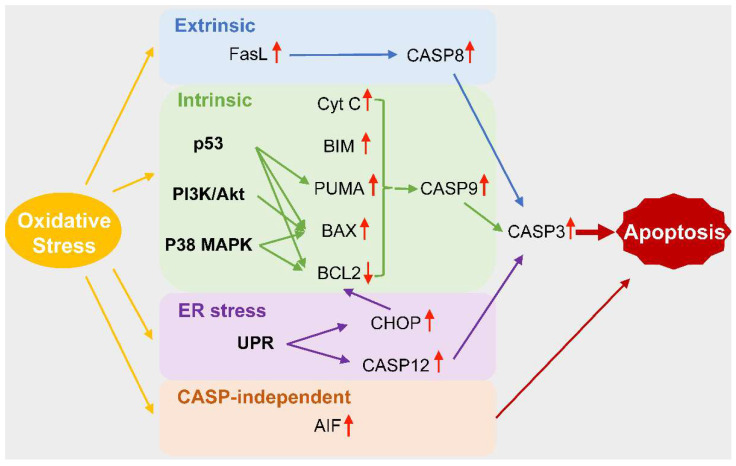
Molecular mechanisms for oxidative stress-triggered podocyte apoptosis. Oxidative stress upregulates FasL to activate the CASP8-mediated extrinsic apoptosis pathway. As for intrinsic apoptosis, p53, PI3K/Akt, and P38 MAPK pathways are activated by oxidative stress, and the expression levels of BAX, PUMA, BIM, and Cyt C are elevated, whereas anti-apoptotic BCL2 is reduced. Whereafter, CASP9 is activated to cleave pro-CASP3 to activate CASP3, which ultimately cleaves cellular components and leads to apoptosis. ER stress is also activated, UPR triggers CHOP- and CASP12-dependent apoptosis, and CHOP activation downregulates BCL2, indicating that intrinsic apoptosis is involved. Moreover, oxidative stress activates CASP-independent apoptosis via upregulating AIF. FasL, Fas ligand; CASP, caspase; Cyt C, cytochrome c; BCL2, B-cell lymphoma-2; BIM, BCL2-interacting mediator of cell death; PUMA, p53-upregulated modulator of apoptosis; BAX, BCL2-associated X; ER, endoplasmic reticulum; UPR, unfolded protein response; CHOP, CCAAT/enhancer-binding protein-homologous protein; and AIF, apoptosis-inducing factor.

Plenty of studies have shown that excessive ROS accumulation triggers podocyte apoptosis both in vivo or in vitro [8,68,100,101,102]. In type 1 and type 2 diabetic models, podocyte apoptosis is correlated with urinary albumin excretion and precedes the loss of the podocyte [8]. The molecular mechanisms for oxidative stress-triggered podocyte apoptosis are summarized in Figure 2.

The ROS-mediated intrinsic apoptosis pathway has been shown to participate in palmitic acid and HG-induced podocyte apoptosis, which can be markedly alleviated by mitotempo and NOX inhibitor apocynin [18,27,84]. Smad3 is activated to upregulate NOX4 after PA treatment [27]. However, extrinsic apoptosis pathway-associated indicators, including the Fas-associated protein with the death domain (FADD) and CASP8, are not changed by an intervention of palmitic acid [18]. Meanwhile, decreased BCL2 expression, increased BAX expression, and the translocation to mitochondria are observed [18,84]. Additionally, more Cyt C is released from the mitochondria and the levels of CASP9 and CASP3 are increased [18,27,84,103].

The ROS-triggered extrinsic apoptosis pathway also has a vital role in the pathogenesis of podocytopathies. AOPP increases the protein level of FOXO3 in the cultured podocyte in a ROS/mTOR-dependent pathway to induce proapoptotic FasL and BIM expression and the sequential upregulation of CASP3, leading to death receptor mediated apoptosis [104].

Mitogen-activated protein kinases (MAPKs) belong to the serine/threonine kinase superfamily that can regulate the activities of organisms, including growth, differentiation, and apoptosis [13]. Among MAPKs, extracellular signal-regulated kinases 1 or 2 (ERK1/2), Jun N-terminal kinase or stress-activated kinases (JNKs/SAPKs), and p38 MAPKs have been widely investigated [13,105]. HG and PA-mediated oxidative stress induces podocyte apoptosis through the activation of the p38 MAPK pathway, which can be attenuated by NOX inhibitor apocynin and antioxidant N-acetylcysteine (NAC) [8,19,106]. In type 1 diabetes and HG treated podocytes, protein kinase A is excited via dopamine 1 receptor activation to upregulate the expression of NOX5, which contributes to oxidative stress and induces the phosphorylation of p38 MAPK and downstream signaling cascades [57]. Moreover, HG also upregulates the expression of NOX4 to induce the phosphorylation of p38 MAPK [83]. The expression of BCL2 is lower in HG-treated podocytes, while BAX, cleaved caspases 3 and 9, are higher when compared with controls, indicating that the mitochondria-mediated apoptosis pathway is activated [57,83,106]. Advanced glycation end-products (AGEs) binding to RAGE can trigger ROS generation to induce podocyte apoptosis through the activation of Forkhead transcription factor 4 (FOXO4) and the p38 MAPK pathway, whereas the inhibition of p38 MAPK or siRNA for FOXO4 abolishes AGE-induced podocyte apoptosis [101].

The phosphatidylinositol 3-kinase (PI3K)/Akt signaling pathway can be activated by various cellular stimuli and regulate cellular functions, such as growth, proliferation, cell cycle, and survival [107]. Previous studies demonstrated that HG increases the level of ROS in the podocyte and induces oxidative stress [8,28]. Meanwhile, ROS downregulate the expression of PI3K and suppress the phosphorylation of Akt [101,108]. Downregulated Akt phosphorylation activates BAX and CASP3 to trigger podocyte apoptosis [108].

p53 is a tumor suppressor transcriptive factor, which responds to various stresses to maintain homeostasis via triggering cell cycle arrest, apoptosis, senescence, and DNA repair [109]. The HG stimulus downregulates AMP-activated protein kinase (AMPK) to upregulate NOX4 expression and activity, which further activates p53 [110,111]. AOPP can also activate NOX to induce the expression of p53 [69,112]. p53 can regulate downstream proapoptotic genes, including the p53-upregulated modulator of apoptosis (PUMA) and BAX [69,110,111,112]. Downregulated BCL2, upregulated CASP3 and CASP9, and DNA fragmentation are consistent with intrinsic apoptosis pathway activation [110,111,112].

ER stress is caused by misfolded or unfolded protein accumulation and an intracellular calcium imbalance, with the unfolded protein response (UPR) determining the cell fate under ER stress [113]. Palmitic acid and AOPP trigger ROS-mediated ER stress to induce podocyte apoptosis [68,100]. Three UPR pathways, including protein kinase-like ER kinase (PERK), activating transcription factor 6 (ATF6), and inositol requiring 1 (IRE1) pathways, are activated to trigger CCAAT/enhancer-binding protein-homologous protein (CHOP)- and CASP12-dependent podocyte apoptosis with BCL2 downregulation involved in CHOP-dependent apoptosis [68,100].

Moreover, the cytosolic apoptosis-inducing factor (AIF) is also increased in the HG-intervened podocyte, which can activate caspase-independent apoptosis [103,114].

Other forms of cell death also take part in ROS-mediated podocyte damage. Pyroptosis has been shown to participate in HIV-associated nephropathy [16]. HIV-transgenic mice exhibit increased mRNA levels and the expression of the NLR family pyrin domain containing 3 (NLRP3), CARD domain containing adaptor protein (ASC), and CASP1 and IL-1β in the kidney, which can be partially inhibited by tempo, suggesting that ROS are crucial to HIV-induced podocyte pyroptosis [16]. Ferroptosis is involved in ROS-mediated podocytopathies as well. Solute carrier family 7 member 11 (SLC7A11) is a member of Xc-system, which transports cysteine and glutamate, and GPX4 is the main endogenous inhibitor of ferroptosis, which reduces the production of phospholipid hydroperoxide [115]. In the HG-stimulated podocyte, the expressions of SLC7A11 and GPX4 are reduced, which can be eliminated by Prdx6 overexpression [17]. Additionally, the protective effect of Prdx6 overexpression on the podocyte can be suppressed by the ferroptosis-inducing agent erastin [17].

Both necrosis and necroptosis are found to participate in the pathogenesis of podocytopathies, such as LN and DN [116,117,118]. However, the evidence for the relationship between oxidative stress, necrosis, and necroptosis in podocytopathies is still lacking, possibly owing to technical restrictions in detecting and validating necrosis and necroptosis in vivo [15].

Since the major researches focus on oxidative stress-mediated apoptosis in DN, further research focusing on the underlying mechanisms of ROS mediating other types of cell death in podocytes exposed to different stimuli under other pathophysiologic conditions is desirable. In conclusion, oxidative stress can trigger podocyte death through a variety of molecular mechanisms. Targeting oxidative stress-mediated podocyte death may be a potential novel therapy for patients with podocytopathies.

## 5. Therapeutic Implications

Since ROS-mediated cell death is of great importance for the pathogenesis of podocytopathies, antioxidants that can protect the podocyte from death are promising for the treatment of patients diagnosed with podocytopathies.

Mitoquinone (MitoQ) is a mitochondria-targeted antioxidant that consists of CoQ10 and triphenyl phosphate. It has been shown to mitigate oxidative stress in various diseases, such as hypoxia-induced pulmonary hypertension and renal ischemia-reperfusion injury [119,120]. In Ang II-treated podocytes, MitoQ suppresses Ang II-induced mitochondrial dysfunction and oxidative stress and reduces podocyte apoptosis [121].

Taurine is an amino sulfonic acid that has been used as an oral supplement for the treatment of disorders, such as congestive heart failure, hypertension, and diabetes mellitus [122,123,124]. Antioxidant activity plays an essential role in the protective effect of taurine [125]. In DN and PA-induced podocytopathies, taurine supply alleviates urinary protein excretion and histopathological lesions [126,127]. Mitochondria ROS generation and podocyte apoptosis induced by HG are attenuated by taurine [127].

Melatonin is a hormone secreted by the brain to maintain the circadian rhythm. Some studies revealed that melatonin can mitigate DN via the inhibition of oxidative stress [128,129]. In Ang II-induced podocyte injury, melatonin reduces podocyte apoptosis and enhances the proliferation capacity of the podocyte [130]. Additionally, the protective effect is associated with inhibited oxidative stress and recovered mitochondrial function [130].

Folic acid is a B vitamin that can reduce plasma Hcy and antagonize the harmful effects generated by it [29,131]. In a spontaneously hypertensive model of rats, a high level of Hcy induces mitochondrial oxidative stress and glomerular damage, whereas even a low level of folic acid could attenuate the glomerular damage caused by Hcy via inhibiting oxidative stress and podocyte apoptosis [29]. Additionally, the antioxidant effect of folic acid may be independent of Hcy [29].

Herbal medicine has been applied in the clinic for thousands of years and is regarded as an alternative treatment for a lot of diseases. A great deal of herbs and herb-derived components are found to have an antioxidative capacity, and have been investigated for the therapeutic effectiveness in podocytopathies. Since oxidative stress-regulated cell death has a significant role in the pathogenesis of podocytopathies, those antioxidative herbs and herb-derived components might improve renal injury via the suppression of the death of the podocyte. Herbs and herb-derived components decrease intracellular ROS via downregulating pro-oxidative enzymes, such as NOX, and elevating mitochondrial respiration chain complex activities [59,83]. The levels of antioxidative enzymes, such as CAT and SOD, and antioxidants, such as GSH, are augmented as well [81,132]. ROS-mediated apoptotic pathways, including PI3K/Akt, p38 MAPK, and p53 pathways are found to be regulated by herbs and herb-derived components [83,108,111]. Except for anti-apoptotic ability, geniposide, an ingredient of Gardenia jasminoides Ellis, can alleviate podocyte injury and inhibit the development of DN via the suppression of pyroptosis [133]. In Table 1, we summarize the antioxidative herbs and herb-derived components that were verified to alleviate podocytopathies via the suppression of cell death.

The therapeutic targets of the antioxidants described above are summarized in Table 2.

Considering that oxidative stress plays a detrimental role in podocytopathies, drugs targeting oxidative stress may have potential value for clinical application in patients with podocytopathies. Hence, we summarize those oxidative stress-targeted drugs in clinical trials from ClinicalTrials.gov in Table 3 [160].

## 6. Conclusions

The evidence supports the determination that mitochondrial oxidative stress, driven by excessive stress and harmful stimuli to the podocyte, plays an essential role in the pathogenesis of podocytopathies. Plenty of studies revealed that oxidative stress can mediate the cell death of the podocyte, especially podocyte apoptosis, via many signaling pathways. Remarkably, oxidative stress can also contribute to the development of podocytopathies via other mechanisms, such as cell cycle arrest. Further researches focusing on the relationship between oxidative stress and other types of cell death in the podocyte are needed. Many potential antioxidants are under investigation for the effectiveness in treating podocytopathies. Considering that podocyte injury is the initiation factor of podocytopathies, and oxidative stress and oxidative stress-mediated cell death are consistent mechanisms among podocyte injury under different pathophysiologic conditions, targeted podocyte oxidative stress therapy that can ameliorate cell death might be promising to slow and even halt the progression of podocytopathies.

## Figures and Tables

**Table 1:** A summary of antioxidative herbs and herb-derived components that can protect the podocyte from death.

Herbs/Components	Models of Podocytopathies	Anti-Death Mechanisms	Refs.
Salvia przewalskii	PA-induced rats	Anti-apoptosis	[89]
Berberine	Palmitic acid-cultured podocytes	Anti-apoptosis	[100]
Apigenin	Adriamycin-induced mice	Anti-apoptosis	[132]
Citral	Adriamycin-induced rats	Anti-apoptosis	[134]
Epigallocatechin-3-Gallate	Adriamycin-induced mice	Anti-apoptosis	[135]
Osthole	Adriamycin-induced mice	Anti-apoptosis	[136]
Leonurine	Adriamycin-induced mice	Anti-apoptosis	[137]
Paclitaxel	Palmitate-cultured podocytes	Anti-apoptosis	[138]
Quercetin	Pristane-induced mice	Anti-apoptosis	[81]
Resveratrol	db/db mice STZ-induced mice	Anti-apoptosis	[59,139,140]
Baoshenfang	KK-Ay mice STZ-induced rats	Anti-apoptosis	[83,108]
Icariin	HG-cultured podocytes	Anti-apoptosis	[103]
Tongxinluo	STZ-induced rats	Anti-apoptosis	[106]
Huangqi	STZ-induced mice	Anti-apoptosis	[111]
Geniposide	HFD/STZ-induced mice	Anti-pyroptosis	[133]
Huidouba	Unilateral nephrectomy/STZ-induced rats	Anti-apoptosis	[141]
Salidroside	HG-cultured podocytes	Anti-apoptosis	[142]
Catalpol	KK-Ay/HFD induced mice HG-cultured podocytes AGEs-cultured podocytes	Anti-apoptosis	[143,144]
Loganin	KK-Ay/HFD mice AGEs-cultured podocytes	Anti-apoptosis	[144]
Astragaloside IV	STZ-induced rats	Anti-apoptosis	[145]
Luteolin	HG-cultured podocytes	Anti-apoptosis	[146]
Chrysin	db/db mice	Anti-apoptosis	[147]
Carnosine	STZ-induced rats HG-cultured podocytes	Anti-apoptosis	[148,149]
Forsythoside A	HG-cultured podocytes	Anti-apoptosis	[150]
Ginsenoside Rb1	STZ-induced mice	Anti-apoptosis	[151]
Grape seed procyanidin B2	HG-treated podocytes	Anti-apoptosis	[152]
Green tea polyphenols	Diabetic patients’ plasma-cultured podocytes	Anti-apoptosis	[153]
Hydroxysafflor yellow A	STZ-induced rats	Anti-apoptosis	[154]
Huaiqihuang	HG-treated podocytes	Anti-apoptosis	[155]
Naringin	STZ-induced rats	Anti-apoptosis	[156]
Mogroside IIIE	HG-treated podocytes	Anti-apoptosis	[157]
Rhizoma Polygonum cuspidatum	STZ-induced rats	Anti-apoptosis	[158]
Tetrahydroxy stilbene glucoside	HG-treated podocytes	Anti-apoptosis	[159]

Abbreviations: PA, puromycin aminonucleoside; STZ, streptozotocin; HG, high glucose; HFD, high-fat diet; and AGEs, advanced glycation end-products.

**Table 2:** A summary of the therapeutic targets of antioxidants that can protect the podocyte from death.

**Therapeutic Targets**		**Antioxidants**
Mitochondrial respiration chain	Complex I	Resveratrol [59,139,140]
	Complex III	Resveratrol [59,139,140]
	Complex IV	Chrysin [147]; Forsythoside A [150]
	Cyt C	Resveratrol [59,139,140]; Carnosine [149]
	Quinone	MitoQ [121]
NADPH oxidase		Folic Acid [29]; Resveratrol [59,139,140]; Baoshenfang [83,108]; Huangqi [111]; Epigallocatechin-3-Gallate [135]; Paclitaxel [138]; Tongxinluo [106]; Huidouba [141]; Catalpol [143,144]; Loganin [144]; Forsythoside A [150]; Ginsenoside Rb1 [151]; Naringin [156]
Antioxidant defense systems	SOD	Melatonin [129]; Folic Acid [29]; Resveratrol [59,139,140]; Quercetin [81]; Apigenin [132]; Leonurine [137]; Paclitaxel [138]; Geniposide [133]; Catalpol [143,144]; Loganin [144]; Astragaloside IV [145]; Forsythoside A [150]; Grape Seed Procyanidin B2 [152]; Hydroxysafflor Yellow A [154]; Mogroside IIIE [157]
	CAT	Quercetin [81]; Astragaloside IV [145]; Forsythoside A [150]; Mogroside IIIE [157]
	GPX	Osthole [136]; Geniposide [133]; Grape Seed Procyanidin B2 [152]; Naringin [156]
	GSH	Apigenin [132]

Abbreviations: Complex I, NADH dehydrogenase; Complex III, ubiquinol-cytochrome c reductase; Complex IV, cytochrome c oxidase; Cyt C, cytochrome c; SOD, superoxide dismutase; CAT, catalase; GPX, glutathione peroxidase; and GSH, glutathione.

**Table 3:** A summary of drugs targeting oxidative stress in clinical trials.

Therapeutic Targets		Drugs in Clinical Trials
Mitochondrial respiration chain	Complex IV	S-equol (NCT02142777), Resveratrol (NCT02123121), etc.
	Quinone	MitoQ (NCT02364648), Ubiquinol (NCT02847585), Coenzyme Q10 (NCT01163500), etc.
NADPH oxidase		GKT137831 (NCT02010242, NCT03865927), Pioglitazone (NCT03060772), Januvia (NCT00659711), ImmunAge (NCT02332993), Threalose plus polyphenols (NCT04061070), Apocynin (NCT03680404), etc.
Antioxidant defense systems	SOD	GC4711 (NCT03762031), PC-SOD (NCT03995732), APN201 (NCT01513278), rhSOD (NCT00264186), Glisodin (NCT03878433), Melatonin (NCT02463318), etc.
	CAT	Melatonin (NCT02463318), Oligopin (NCT03260803), etc.
	GPX	N-acetylcysteine (NCT00493727), Curcumin (NCT03475017), Rutin (NCT04955145), Melatonin (NCT01858909), etc.
	GSH	Glutathione (NCT02948673, NCT02948673), etc.
	Other antioxidants	Vitamin E (NCT00384618), Vitamin C (NCT04210453), Vitamin D3 (NCT03931889), L-carnitine (NCT01819701), etc.

Abbreviations: Complex IV, cytochrome c oxidase; SOD, superoxide dismutase; CAT, catalase; GPX, glutathione peroxidase; and GSH, glutathione.
